# *S^3^*CMTF: Fast, accurate, and scalable method for incomplete coupled matrix-tensor factorization

**DOI:** 10.1371/journal.pone.0217316

**Published:** 2019-06-28

**Authors:** Dongjin Choi, Jun-Gi Jang, U Kang

**Affiliations:** 1 School of Computational Science and Engineering, Georgia Institute of Technology, Atlanta, Georgia, United States of America; 2 Department of Computer Science and Engineering, Seoul National University, Seoul, Republic of Korea; Mayo Clinic Arizona, UNITED STATES

## Abstract

How can we extract hidden relations from a tensor and a matrix data simultaneously in a fast, accurate, and scalable way? Coupled matrix-tensor factorization (CMTF) is an important tool for this purpose. Designing an accurate and efficient CMTF method has become more crucial as the size and dimension of real-world data are growing explosively. However, existing methods for CMTF suffer from lack of accuracy, slow running time, and limited scalability. In this paper, we propose *S*^3^CMTF, a fast, accurate, and scalable CMTF method. In contrast to previous methods which do not handle large sparse tensors and are not parallelizable, *S*^3^CMTF provides parallel sparse CMTF by carefully deriving gradient update rules. *S*^3^CMTF asynchronously updates partial gradients without expensive locking. We show that our method is guaranteed to converge to a quality solution theoretically and empirically. *S*^3^CMTF further boosts the performance by carefully storing intermediate computation and reusing them. We theoretically and empirically show that *S*^3^CMTF is the fastest, outperforming existing methods. Experimental results show that *S*^3^CMTF is up to 930× faster than existing methods while providing the best accuracy. *S*^3^CMTF shows linear scalability on the number of data entries and the number of cores. In addition, we apply *S*^3^CMTF to Yelp rating tensor data coupled with 3 additional matrices to discover interesting patterns.

## Introduction

Given a tensor data, and related matrix data, how can we analyze them efficiently? Tensors (i.e., multi-dimensional arrays) and matrices are natural representations for various real world high-order data [1, 2, 3]. For instance, an online review site Yelp provides rich information about users (name, friends, reviews, etc.), or businesses (name, city, Wi-Fi, etc.). One popular representation of such data includes a 3-way rating tensor with (user ID, business ID, time) triplets and an additional friendship matrix with (user ID, user ID) pairs. Coupled matrix-tensor factorization (CMTF) is an effective tool for joint analysis of coupled matrices and a tensor. The main purpose of CMTF is to integrate matrix factorization [4] and tensor factorization [5] to efficiently extract the factor matrices of each mode. The extracted factors have many useful applications such as latent semantic analysis [6, 7, 8], recommendation systems [9, 10], network traffic analysis [11], and completion of missing values [12, 13, 14].

However, existing CMTF methods do not provide good performance in terms of time, accuracy, and scalability. CMTF-Tucker-ALS [15], a method based on Tucker decomposition [16], has a limitation that it is only applicable for dense data and not parallelizable. For sparse real-world data, it assumes empty entries as zero and outputs highly skewed results which lead to high reconstruction error. Moreover, CMTF-Tucker-ALS does not scale to large data because it suffers from high memory requirement caused by *M-bottleneck problem* [17]. CMTF-OPT [12] is a CMTF method based on CANDECOMP/PARAFAC (CP) decomposition [18]. SDF [19] provided Quasi-Newton and nonlinear least squares optimization techniques for general coupled factorization problems where factors may have certain structures as Toeplitz, orthogonal and nonnegative. CMTF-Tucker-ALS and CMTF-OPT undergo high reconstruction error since the former is not applicable to sparse data, and the latter focuses only on CP model and thus cannot be generalized to the Tucker model. Furthermore, both methods are sequential and hard to take benefit of multi-core parallelization.

**Fig 1:**
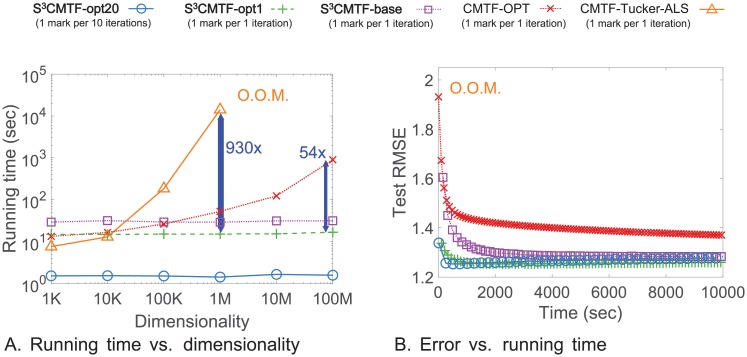
Comparison of our proposed *S*^3^CMTF and the existing methods. (a) For a fixed number of nonzeros, *S*^3^CMTF takes constant time as dimensionality grows, while existing methods become slower. Our sequential method *S*^3^CMTF-opt1 is 930× and 54× faster than CMTF-OPT and CMTF-Tucker ALS, respectively. (b) *S*^3^CMTF-opt20 shows the best convergence rate and accuracy on real world Yelp dataset. CMTF-Tucker-ALS shows O.O.M. in both experiments. (O.O.M.: out of memory error).

In this paper, we propose *S*^3^CMTF (**S**parse, lock-free **S**GD based, and **S**calable CMTF), a CMTF method which resolves the problems of previous methods. *S*^3^CMTF provides parallel, sparse CMTF based on Tucker factorization unlike previous methods which do not support sparse tensors or cannot be parallelized. We also show that asynchronously parallel stochastic gradient descent (SGD) is useful for *S*^3^CMTF in multi-core shared memory systems without expensive locking. *S*^3^CMTF further boosts the performance by storing intermediate computation and reusing them. Table 1 shows the comparison of *S*^3^CMTF and other existing methods. The main contributions of our study are as follows:

- **Algorithm**: We propose *S*^3^CMTF, a coupled tensor-matrix factorization algorithm for matrix-tensor joint datasets. *S*^3^CMTF is designed to efficiently extract factors from the joint datasets by taking advantage of sparsity, exploiting intermediate data. We propose a method which resolves conflicts of parallelization and leads to a solution with guaranteed convergence.
- **Performance**: *S*^3^CMTF shows the best performance on accuracy, speed, and scalability. *S*^3^CMTF runs up to **930× faster** and is more scalable than existing methods as shown in Fig 1A. For real-world datasets, *S*^3^CMTF converges faster to the better optimum as shown in Fig 1B.
- **Discovery**: Applying *S*^3^CMTF on Yelp review dataset with a 3-mode tensor (user, business, time) coupled with 3 additional matrices ((user, user), (business, category), and (business, city)), we observe interesting patterns and clusters of businesses and suggest a process for personal recommendation.

**Table 1:** Comparison of our proposed *S*^3^CMTF and the existing CMTF methods. *S*^3^CMTF outperforms all other methods in terms of time, accuracy, scalability, memory usage, and parallelizability.

Method	Time	Accuracy	Scalability	Memory	Parallel
CMTF-Tucker-ALS	slow	low	low	high	no
CMTF-OPT	slow	low	low	high	no
**S^3^CMTF-base**	fast	**high**	**high**	**lower**	**yes**
**S^3^CMTF-opt**	**faster**	**high**	**high**	low	**yes**

## Preliminaries and related works

In this section, we describe preliminaries for tensor and coupled matrix-tensor factorization. We list all symbols used in this paper in Table 2.

**Table 2:** Table of symbols.

Symbol	Definition
X	input tensor
G	core tensor
*N*	order (number of modes) of the input tensor
*I*_*n*_	dimensionality of *n*-th mode of input tensor X
*J*_*n*_	dimensionality of *n*-th mode of core tensor G
*α*	a tensor index (*i*_1_ *i*_2_⋯*i*_*N*_)
*x*_*α*_	the entry of X with index *α*
**X**_(*n*)_	mode-*n* matricization of a tensor
**U**^(*n*)^	*n*-th factor matrix of X
{**U**}	set of all factor matrices of X
$u_{i}^{\left(n\right)}$	the *i*-th row vector of **U**^(*n*)^
{**u**}_*α*_	ordered set of row vectors $\left\{u_{i_{1}}^{\left(1\right)},u_{i_{2}}^{\left(2\right)},\ldots ,u_{i_{N}}^{\left(N\right)}\right\}$
${\left\{u\right\}}_{\alpha }^{⊤}$	ordered set of column vectors $\left\{u_{i_{1}}^{(1)⊤},u_{i_{2}}^{(2)⊤},\ldots ,u_{i_{N}}^{(N)⊤}\right\}$
$u_{ij}^{\left(n\right)}$	entry of **U**^(*n*)^ with index (*i*, *j*)
**Y**	coupled matrix
*β*	a matrix index *k*_1_*k*_2_
*y*_*β*_	the entry of **Y** with index *β*
**V**	factor matrix for the coupled matrix **Y**
**v**_*k*_	the *k*-th row vector of **V**
$\Omega_{X}$	observed index set of X
$\Omega_{X}^{n,i}$	subset of $\Omega_{X}$ having *i* as the *n*-th index

### Tensor

A tensor is a multi-dimensional array. Each ‘dimension’ of a tensor is called *mode* or *way*. The length of each mode is called ‘dimensionality’ and denoted by *I*_1_, ⋯, *I*_*N*_. In this paper, an *N*-mode or *N*-way tensor is denoted by the boldface Euler script capital (e.g. $X\in R^{I_{1}\times I_{2}\times \ldots \times I_{N}}$), and matrices are denoted by boldface capitals (e.g. **A**). *x*_*α*_ and *a*_*β*_ denote the entry of X and **A** with indices *α* and *β*, respectively.

We describe tensor operations used in this paper. A mode-*n* fiber is a vector which has fixed indices except for the *n*-th index in a tensor. The mode-*n* matrix product of a tensor $X\in R^{I_{1}\times I_{2}\times \ldots \times I_{N}}$ with a matrix $A\in R^{J\times I_{n}}$ is denoted by $X\times_{n}A$ and has the size of *I*_1_×⋯*I*_*n*−1_×*J*×*I*_*n*+1_ ⋯ × *I*_*N*_. It is defined as:

$$\begin{array}{c} {\left(X\times_{n}A\right)}_{i_{1}\cdots i_{n-1}ji_{n+1}\cdots i_{N}}=\sum_{i_{n}=1}^{I_{n}}x_{i_{1}i_{2}\cdots i_{N}}a_{ji_{n}}, \end{array}$$ (1)

where $a_{ji_{n}}$ is the (*j*, *i*_*n*_)-th entry of **A**. For brevity, we use the following shorthand notation for multiplication on every mode as in [20]:

$$\begin{array}{c} X\times \left\{A\right\}≔X\times_{1}A^{\left(1\right)}\times_{2}A^{\left(2\right)}\cdots \times_{N}A^{\left(N\right)}, \end{array}$$ (2)

where {**A**} denotes the ordered set {**A**^(**1**)^, **A**^(2)^, ⋯, **A**^(*N*)^}.

We use the following notation for multiplication on every mode except *n*-th mode.

$$\begin{array}{c} X\times_{-n}\left\{A\right\}≔X\times_{1}A^{\left(1\right)}\cdots \times_{n-1}A^{\left(n-1\right)}\times_{n+1}A^{\left(n+1\right)}\cdots \times_{N}A^{\left(N\right)}. \end{array}$$

We examine the case that an ordered set of row vectors {**a**^(**1**)^, **a**^(**2**)^, ⋯, **a**^(**N**)^}, denoted by {**a**}, is multiplied to a tensor X. First, consider the multiplication for every corresponding mode. By Eq (1),

$$\begin{array}{c} X\times \left\{a\right\}=\sum_{i_{1}=1}^{I_{1}}\sum_{i_{2}=1}^{I_{2}}\cdots \sum_{i_{N}=1}^{I_{N}}x_{i_{1}i_{2}\cdots i_{N}}a_{i_{1}}^{\left(1\right)}a_{i_{2}}^{\left(2\right)}\cdots a_{i_{N}}^{\left(N\right)}, \end{array}$$

where $a_{k}^{\left(m\right)}$ denotes the *k*-th element of **a**^(*m*)^. Then, consider the multiplication for every mode except *n*-th mode. Such multiplication results in a vector of length *I*_*n*_. The *k*-th entry of the vector is

$$\begin{array}{c} {}[X\times_{-n}\left\{a\right\}{]}_{k}=\sum_{\forall \alpha \in \Omega_{X}^{n,k}}x_{\alpha }a_{i_{1}}^{\left(1\right)}\cdots a_{i_{n-1}}^{\left(n-1\right)}a_{i_{n+1}}^{\left(n+1\right)}\cdots a_{i_{N}}^{\left(N\right)}, \end{array}$$ (3)

where $\Omega_{X}^{n,k}$ denotes the index set of X having its *n*-th index as *k*. *α* = (*i*_1_ *i*_2_⋯*i*_*N*_) denotes the index for an entry.

### Tucker decomposition

Tucker decomposition is one of the most popular tensor factorization models. Tucker decomposition factorizes an *N*-mode tensor $X\in R^{I_{1}\times I_{2}\times \ldots \times I_{N}}$ into a core tensor $G\in R^{J_{1}\times J_{2}\times \ldots \times J_{N}}$ and factor matrices $U^{\left(1\right)}\in R^{I_{1}\times J_{1}},U^{\left(2\right)}\in R^{I_{2}\times J_{2}},\ldots ,U^{\left(N\right)}\in R^{I_{N}\times J_{N}}$ satisfying

$$\begin{array}{c} X\approx \tilde{X}=G\times_{1}U^{\left(1\right)}\times_{2}U^{\left(2\right)}\cdots \times_{N}U^{\left(N\right)}=G\times \left\{U\right\}. \end{array}$$

Element-wise formulation of Tucker model is

$$\begin{array}{c} \begin{array}{cc} x_{\alpha }\approx {\tilde{x}}_{\alpha } & =\sum_{j_{1}=1}^{J_{1}}\sum_{j_{2}=1}^{J_{2}}\cdots \sum_{j_{N}=1}^{J_{N}}g_{j_{1}j_{2}\cdots j_{N}}u_{i_{1}j_{1}}^{\left(1\right)}u_{i_{2}j_{2}}^{\left(2\right)}\cdots u_{i_{N}j_{N}}^{\left(N\right)}\\ & =G\times_{1}u_{i_{1}}^{\left(1\right)}\times_{2}u_{i_{2}}^{\left(2\right)}\cdots \times_{N}u_{i_{N}}^{\left(N\right)}≔G\times {\left\{u\right\}}_{\alpha }, \end{array} \end{array}$$ (4)

where *α* is a tensor index (*i*_1_*i*_2_⋯*i*_*N*_), and $u_{i_{n}}^{\left(n\right)}$ denotes the *i*_*n*_-th row of factor matrix **U**^(*n*)^. {**u**}_*α*_ denotes the set of factor rows $\left\{u_{i_{1}}^{\left(1\right)},u_{i_{2}}^{\left(2\right)},\ldots ,u_{i_{N}}^{\left(N\right)}\right\}$. The core tensor G indicates the relation between the factors in Tucker formulation. When the core tensor size is restricted as *J*_1_ = *J*_2_ = ⋯ = *J*_*N*_ and the core tensor structure is hyper-diagonal, it is equivalent to CANDECOMP/PARAFAC (CP) decomposition. Orthogonality constraint can optionally be imposed to the Tucker decomposition by forcing the factor matrices to have orthonormal columns (e.g. **U**^(*n*)*T*^ **U**^(*n*)^ = **I** for *n* = 1, ⋯, *N* where **I** is an identity matrix).

### Coupled matrix-tensor factorization

Coupled matrix-tensor factorization (CMTF) is proposed for joint factorization of a tensor and matrices. CMTF integrates matrix factorization and tensor factorization.

**Definition 1**. ***(Coupled Matrix-Tensor Factorization)*** *Given an N-mode tensor* $X\in R^{I_{1}\times \ldots \times I_{N}}$ *and a matrix* $Y\in R^{I_{c}\times K}$ *where c is the coupled mode*, $X\approx \tilde{X}=G\times \left\{U\right\}$, *and* $Y\approx \tilde{Y}=U^{\left(c\right)}V^{⊤}$ *are the coupled matrix-tensor factorization*. $U^{\left(c\right)}\in R^{I_{c}\times J_{c}}$ *is the c-th mode factor matrix, and* $V\in R^{K\times J_{c}}$ *denotes the factor matrix for the coupled matrix*. *Finding the factor matrices and core tensor for CMTF is equivalent to solving*

$$\begin{array}{c} \underset{U^{\left(1\right)},\cdots ,U^{\left(N\right)},V,G}{\arg\min}{\left\|X-G\times \left\{U\right\}\right\|}^{2}+{\left\|Y-U^{\left(c\right)}V^{⊤}\right\|}^{2}, \end{array}$$ (5)

*where* ‖ • ‖ *denotes the Frobenius norm*.

Various methods have been proposed to efficiently solve the CMTF problem. An alternating least squares (ALS) method CMTF-Tucker-ALS [15] was proposed. CMTF-Tucker-ALS is based on Tucker-ALS (HOOI) [21] which is a popular method for fitting the Tucker model. Tucker-ALS suffers from a crucial intermediate memory-bottleneck problem known as *M-bottleneck problem* [17] that arises from materialization of a large dense tensor $X\times_{-n}{\left\{U\right\}}^{⊤}$ as intermediate data where ${\left\{U\right\}}^{⊤}=\left\{U^{(1)⊤},U^{(2)⊤},\ldots ,U^{(N)⊤}\right\}$. Generalized coupled tensor factorization frameworks [22, 23] have been proposed, and they propose multiplicative methods for non-negative factorization. SDF [19] provided Quasi-Newton and nonlinear least squares optimization techniques for general coupled factorization problems where factors may have certain structures as Toeplitz, orthogonal and nonnegative. A Bayesian method [24] has been proposed. It suggests a generative model for tensor factorization and gets parameters with Gibbs sampling method. Most methods for CMTF use CP decomposition model for $\tilde{X}$ where *J*_1_ = *J*_2_ = ⋯ = *J*_*N*_ and the core tensor G is hyper-diagonal [12, 25, 26, 27, 28, 19]. CMTF-OPT [12] is a representative algorithm for this problem which uses nonlinear conjugate gradient descent method to find factors. HaTen2 [26, 29], and SCouT [25] propose distributed methods for CMTF using CP decomposition model based on the MapReduce framework. Turbo-SMT [27] provides a time-boosting technique for CP-based CMTF methods.

Note that Eq (5) requires all data entries of X and **Y** to be observed. Unobserved values are set to zeros when X and **Y** are sparse, which results in low accuracy. However, most real world data set shows high sparsity. For example, the density of real world tensor we use for experiments vary from 10^−7^ to 10^−4^. For this reason above methods show low accuracy for real-world sparse data; what we focus on this paper is solving CMTF for sparse data.

**Definition 2**. ***(Sparse CMTF)*** *When* X *and* **Y** *are sparse, sparse CMTF aims to find factors only considering the observed entries*. *Let* $W^{\left(1\right)}$ *indicates the observed entries of* X *such that*

$$\begin{array}{ccc} w_{\alpha }^{\left(1\right)}=\{\begin{array}{cc} 1 & if\;x_{\alpha }\;is\;observed\\ 0 & if\;x_{\alpha }\;is\;unobserved \end{array} & & \text{,}\;for\;\forall \alpha \in \Omega_{X}. \end{array}$$

*Let* **W**^(2)^ *indicates the observed entries of* **Y** *analogously*. *We modify* Eq (5) *as*

$$\begin{array}{c} \underset{U^{\left(1\right)},\cdots ,U^{\left(N\right)},V,G}{\arg\text{min}}\|W^{\left(1\right)}{*\left(X-G\times \left\{U\right\}\right)\|}^{2}+{\left\|W^{\left(2\right)}*\left(Y-U^{\left(c\right)}V^{⊤}\right)\right\|}^{2}, \end{array}$$ (6)

*where* * *denotes the Hadamard product (element-wise product)*.

CMTF-Tucker-ALS does not support sparse CMTF since it calculates a singular vector of full and dense matrix. CMTF-OPT provides single machine approach for sparse CMTF for CP model, and CDTF [30] and FlexiFaCT [28] provide distributed methods for sparse CMTF for CP model. Note that all existing methods are based on CP model. Our method is for more general setting, Tucker decomposition, and also easily applied to CP model.

## Proposed method

### Overview

*S*^3^CMTF provides an algorithm for the joint factorization of Tucker decomposition. The major challenge of parallel Tucker decomposition is to avoid the race condition, and design an efficient algorithm for updating factors.

In this section, we describe *S*^3^CMTF (Sparse, lock-free SGD based, and Scalable CMTF), our proposed method for fast, accurate, and scalable CMTF. Our purpose is to minimize the number of race conditions with probabilistic guarantee by exploiting problem characteristic and minimize calculations by exploiting intermediate data.

**Fig 2:**
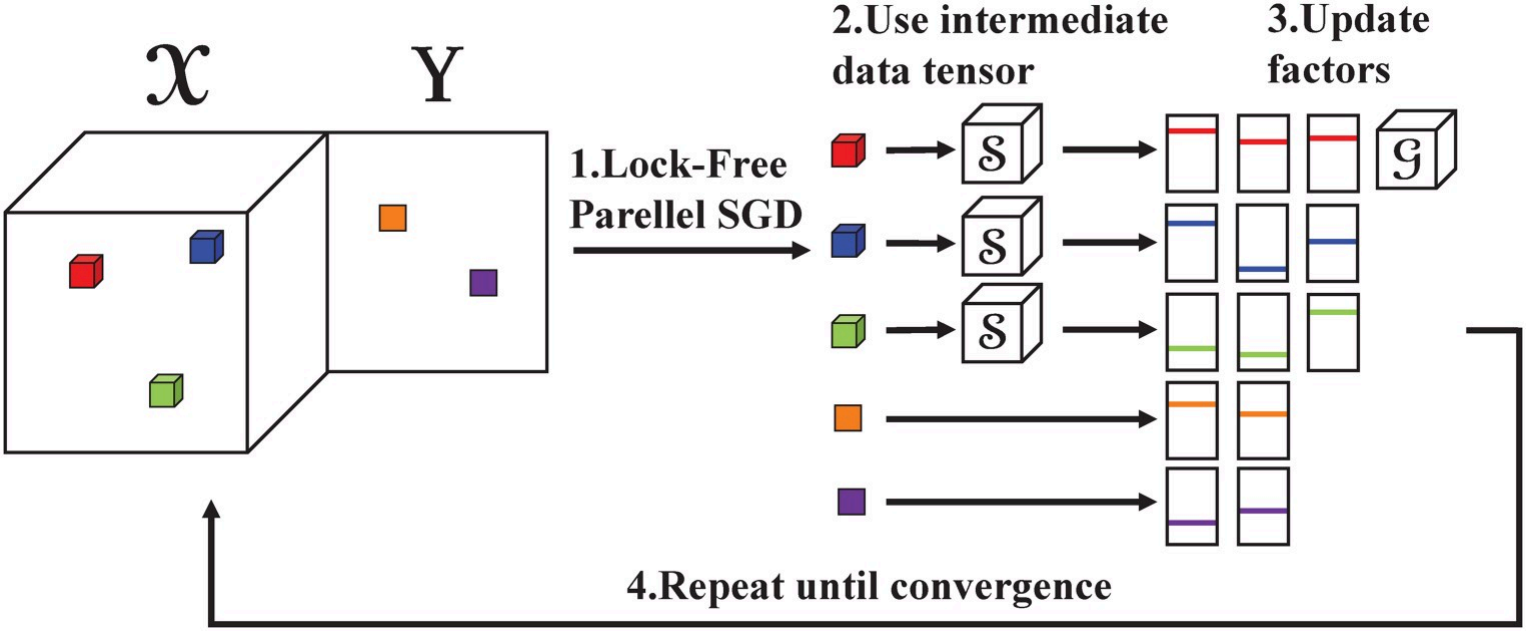
The scheme for *S*^3^CMTF.

We first propose a lock-free parallel method *S*^3^CMTF-base; then, we propose a time-improved version *S*^3^CMTF-opt. Fig 2 shows the overall scheme of *S*^3^CMTF. *S*^3^CMTF-base employs asynchronous parallel SGD for the parallel update with proper workload distribution, and *S*^3^CMTF-opt further improves the speed of *S*^3^CMTF-base by exploiting intermediate data and reusing them.

### Objective function & gradient

We discuss the improved formulation of the sparse CMTF problem defined in Definition 2. For simplicity, we consider the case that one matrix $Y\in R^{I_{c}\times K}$ is coupled to the *c*-th mode of a tensor $X\in R^{I_{1}\times \ldots \times I_{N}}$. Naive calculation of Eq (6) takes excessive time and memory since it includes materialization of dense tensor G×{U}. Therefore, we re-formulate the new CMTF objective function *f* to exploit the sparsity of data and add regularization. *f* is the weighted sum of two functions *f*_*t*_ and *f*_*m*_ which are element-wise sums of squared reconstruction error and regularization terms of tensor X and matrix **Y**, respectively.

$$\begin{array}{c} f=\frac{1}{2}f_{t}+\frac{\lambda_{m}}{2}f_{m}, \end{array}$$ (7)

where λ_*m*_ is a balancing factor of the two functions.

$$\begin{array}{c} f_{t}=[\sum_{\forall \alpha \in \Omega_{X}}(x_{\alpha }-\left(G\times {\left\{u\right\}}_{\alpha }\right){)}^{2}]+\lambda_{reg}({\left\|G\right\|}^{2}+\sum_{n=1}^{N}{\left\|U^{\left(n\right)}\right\|}^{2}), \end{array}$$

where *α* = (*i*_1_⋯*i*_*N*_), $\Omega_{X}$ is the observable index set of X, and λ_*reg*_ denotes the regularization parameter for factors. We rewrite the equation so that it is amenable to SGD update:

$$\begin{array}{c} f_{t}=\sum_{\forall \alpha \in \Omega_{X}}[(x_{\alpha }-\left(G\times {\left\{u\right\}}_{\alpha }\right){)}^{2}+\frac{\lambda_{reg}}{|\Omega_{X}|}{\left\|G\right\|}^{2}+\lambda_{reg}\sum_{n=1}^{N}\frac{\|u_{i_{n}}^{\left(n\right)}{\|}^{2}}{|\Omega_{X}^{n,i_{n}}|}], \end{array}$$

where *α* = (*i*_1_⋯*i*_*N*_). Note that $\Omega_{X}^{n,i_{n}}$ is the subset of $\Omega_{X}$ having *i*_*n*_ as the *n*-th index. Now we formulate *f*_*m*_, the sum of squared errors of coupled matrix and regularization term corresponding to the coupled matrix.

$$\begin{array}{c} f_{m}=\sum_{\forall \beta =\left(j_{1}j_{2}\right)\in \Omega_{Y}}[(y_{\beta }-u_{j_{1}}^{\left(c\right)}v_{j_{2}}^{⊤}{)}^{2}+\frac{\lambda_{reg}}{|\Omega_{Y}^{2,j_{2}}|}{\left\|v_{j_{2}}\right\|}^{2}]. \end{array}$$

We calculate the gradient of *f* (Eq (7)) with respect to factors and core for stochastic gradient descent update. Consider that we pick one index $\alpha =\left(i_{1}\ldots i_{N}\right)\in \Omega_{X}$ and matrix index *β* = (*j*_1_*j*_2_) ∈ Ω_**Y**_. We calculate the corresponding partial derivatives of *f* with respect to the factors and the core tensor as follows.

$$\begin{array}{c} {\frac{\partial f}{\partial u_{i_{n}}^{\left(n\right)}}|}_{\alpha }=-(x_{\alpha }-\left(G\times {\left\{u\right\}}_{\alpha }\right))[{\left(G\times_{-n}{\left\{u\right\}}_{\alpha }\right)}_{\left(n\right)}{]}^{⊤}+\frac{\lambda_{reg}}{|\Omega_{X}^{n,i_{n}}|}u_{i_{n}}^{\left(n\right)}, \end{array}$$ (8a)

$$\begin{array}{c} {\frac{\partial f}{\partial G}|}_{\alpha }=-(x_{\alpha }-\left(G\times {\left\{u\right\}}_{\alpha }\right))\times {\left\{u\right\}}_{\alpha }^{⊤}+\frac{\lambda_{reg}}{|\Omega_{X}|}G, \end{array}$$ (8b)

$$\begin{array}{c} {\frac{\partial f}{\partial u_{j_{1}}^{\left(c\right)}}|}_{\beta }=-\lambda_{m}\left(y_{\beta }-u_{j_{1}}^{\left(c\right)}v_{j_{2}}^{⊤}\right)v_{j_{2}}, \end{array}$$ (8c)

$$\begin{array}{c} {\frac{\partial f}{\partial v_{j_{2}}}|}_{\beta }=-\lambda_{m}\left(y_{\beta }-u_{j_{1}}^{\left(c\right)}v_{j_{2}}^{⊤}\right)u_{j_{1}}^{\left(c\right)}+\frac{\lambda_{m}\lambda_{reg}}{|\Omega_{Y}^{2,j_{2}}|}v_{j_{2}}. \end{array}$$ (8d)

Note that our formulated coupled matrix-tensor factorization model is easily generalized to the case that multiple matrices are coupled to a tensor. We couple multiple matrices to a tensor for experiments in Sections for experiments and discovery.

### Multi-core parallelization

How can we parallelize the SGD updates for CMTF in multiple cores? In CMTF, SGD is hard to be parallelized without conflicts since each update may suffer from memory conflicts by attempting to write the core tensor G to memory concurrently [31]. One solution for this problem is memory locking and synchronization. However, there are lots of overhead associated with locking. Therefore, we use lock-free strategy to parallelize *S*^3^CMTF. We develop a parallel update scheme for *S*^3^CMTF by adopting HOGWILD! update scheme [32]. For any SGD problem, a hypergraph is induced where its nodes represent parameters and edges represent the set of parameters related to a data point.

**Fig 3:**
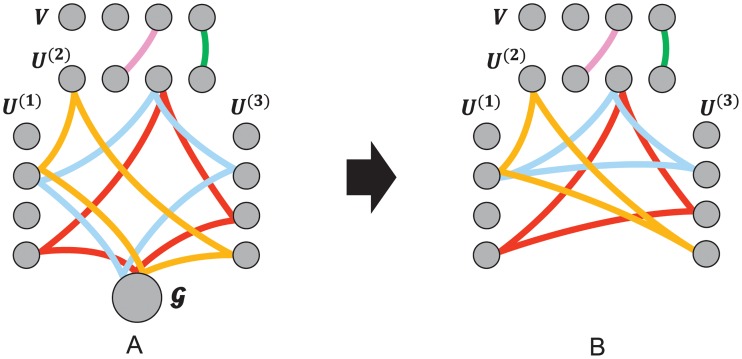
Example hypergraphs induced by *S*^3^CMTF objective function (Eq (7)). A matrix **Y** is coupled to the second mode of X with a coupled factor matrix **V**. Each node represents a factor row or the core tensor. Each hyperedge includes corresponding factors to an SGD update. (a) Induced hypergraph with the core tensor. Every hyperedge corresponding to tensor entries includes G. (b) Induced hypergraph without core tensor. The graph has sparse structure as every node is shared by only few hyperedges.

**Definition 3**. ***(Induced Hypergraph)*** *The objective function in* Eq (7) *induces a hypergraph G* = (*V*, *E*) *whose nodes represent factor rows and the core tensor*. *Each entry of* X *and* **Y** *induces a hyperedge e* ∈ *E consisting of corresponding factor rows or core tensor*. Fig 3A *shows an example induced graph of S*^3^CMTF.

Lock-free parallel updates often converge nearly linearly for a sparse SGD problem in which conflicts between different updates rarely occur [32]. However, in CMTF with Tucker formulation, every update of tensor entries includes the core tensor G as shown in Fig 3A. We allocate the update of the core tensor G to one dedicated CPU core and increase the step size by the number to keep the expected step size unchanged, which leads to line 7 of Algorithm 1 described in the next section. Then we obtain a new induced hypergraph in Fig 3B. Previous induced hypergraph (Fig 3A) implies that every factor update (red, blue, and orange hyperedges) is in conflict with each other on updating the core tensor, resulting to unexpected behaviors. In contrast, the new induced hypergraph shows that the update of factors is independent of that of the core tensor.

Note that our problem with this induced hypergraph is a general case of matrix completion problem in [32] which provides convergence guarantee of lock-free parallelism; each edge in our hypergraph entails *N* vertices, while that in [32] entails only 2 vertices.

**Algorithm 1** *S*^3^CMTF-base

**Require**: Tensor $X\in R^{I_{1}\times \ldots \times I_{N}}$, rank (*J*_1_, ⋯, *J*_*N*_), number of parallel cores *P*, initial learning rate *η*_0_, decay rate *μ*, coupled mode *c*, and coupled matrix $Y\in R^{I_{c}\times K}$

**Emsure**: Core tensor $G\in R^{J_{1}\times \ldots \times J_{N}}$, factor matrices **U**^(1)^, ⋯, **U**^(*N*)^, **V**

1: Initialize G, $U^{\left(n\right)}\in R^{I_{n}\times J_{n}}$ for *n* = 1, ⋯, *N*, and **V** randomly

2: **repeat**

3:  **for** $\forall \alpha =\left(i_{1}\ldots i_{N}\right)\in \Omega_{X}$, ∀*β* = (*j*_1_*j*_2_) ∈ Ω_**Y**_ in random order **do in parallel**

4:   **if** *α* is picked **then**

5:    ($\frac{\partial f}{\partial u_{i_{1}}^{\left(1\right)}}$,⋯,$\frac{\partial f}{\partial u_{i_{N}}^{\left(N\right)}}$,$\frac{\partial f}{\partial G}$) ←*compute_gradient*(*α*,*x*_*α*_,G)

6:    $u_{i_{n}}^{\left(n\right)}\leftarrow u_{i_{n}}^{\left(n\right)}-\eta_{t}\frac{\partial f}{\partial u_{i_{n}}^{\left(n\right)}}$, (for *n* = 1, ⋯, *N*)

7:    $G\leftarrow G-\eta_{t}P\frac{\partial f}{\partial G}$ (executed by one dedicated CPU core)

8:   **end if**

9:   **if** *β* is picked **then**

10:    ${\tilde{y}}_{\beta }\leftarrow u_{j_{1}}^{c}v_{j_{2}}^{⊤}$, $\frac{\partial f}{\partial u_{j_{1}}^{\left(c\right)}}\leftarrow -\lambda_{m}\left(y_{\beta }-{\tilde{y}}_{\beta }\right)v_{j_{2}}$

11:    $\frac{\partial f}{\partial v_{j_{2}}}\leftarrow -\lambda_{m}\left(y_{\beta }-{\tilde{y}}_{\beta }\right)u_{j_{1}}^{\left(c\right)}+\frac{\lambda_{m}\lambda_{reg}}{|\Omega_{Y_{2,j_{2}}}|}v_{j_{2}}$

12:    $u_{j_{1}}^{\left(c\right)}\leftarrow u_{j_{1}}^{\left(c\right)}-\eta_{t}\frac{\partial f}{\partial u_{j_{1}}^{\left(c\right)}}$, $v_{j_{2}}\leftarrow v_{j_{2}}-\eta_{t}\frac{\partial f}{\partial v_{j_{2}}}$

13:   **end if**

14:  **end for**

15:  *η*_*t*_ = *η*_0_(1 + *μt*)^−1^

16: **until** convergence conditions are satisfied

17: **for** *n* = 1, …, *N* **do**

18:  **Q**^(*n*)^,**R**^(*n*)^ ← QR decomposition of **U**^(*n*)^

19:  **U**^(*n*)^ ← **Q**^(*n*)^, $G\leftarrow G\times_{n}R^{\left(n\right)}$

20: **end for**

21: $V\leftarrow VR^{(c)⊤}$

22: **return** G, **U**^(1)^, ⋯, **U**^(*N*)^, **V**

### S^3^CMTF-base

We present our method, *S*^3^CMTF-base, combination of the aforementioned techniques. *S*^3^CMTF-base solves the sparse CMTF problem by parallel SGD techniques explained above. Algorithm 1 shows the procedure of *S*^3^CMTF-base. In the beginning, *S*^3^CMTF-base initializes factor matrices and the core tensor randomly (line 1 of Algorithm 1). The outer loop (lines 2-16) repeats until the factor variables converge. The inner loop (lines 3-14) is performed by several cores in parallel. In each inner loop, *S*^3^CMTF-base selects an index which belongs to $\Omega_{X}$ or Ω_**Y**_ in random order (line 3). If a tensor index *α* is picked, then the algorithm calculates the partial gradients of corresponding factor rows using *compute_gradient* (Algorithm 2) in line 5, and updates factor row vectors (line 6). Core tensor G is updated by one dedicated CPU core (line 7). Note that if line 7 is run by multiple cores, a core may interrupt another core’s update of G by overwriting the gradient $\frac{\partial f}{\partial G}$, which leads to unexpected update of G and hinders convergence; thus, we eliminate the possibility of such conflict by allocating update of G to the dedicated CPU core. The update of line 7 is done independently by the dedicated CPU core, but concurrently with gradient calculation (line 5) and factor updates (line 6) of other CPU cores. The number *P* of cores is multiplied to the gradient to compensate for the one-core update so that SGD uses the same expected learning rate for all the parameters. If a coupled matrix index *β* is picked, then the gradient update is performed on corresponding factor row vectors (lines 9-13). At the end of the outer loop, the learning rate *η*_*t*_ of the *t*-th iteration is monotonically decreased [33]. (line 15). QR decomposition is applied on factors to satisfy orthogonality constraint of factor matrices (lines 17-20). QR decomposition of **U**^(*n*)^ generates **Q**^(*n*)^, an orthogonal matrix of the same size as **U**^(*n*)^, and a square matrix $R^{\left(n\right)}\in R^{J_{n}\times J_{n}}$. Substituting **U**^(*n*)^ by **Q**^(*n*)^ (line 19) and G by $G\times_{1}R^{\left(1\right)}\ldots \times_{N}R^{\left(N\right)}$ (after *N*-th execution of line 19) result in orthogonal factors with equivalent factorization quality [5]. In the same manner, we substitute **V** by ${\text{VR}}^{(c)⊤}$ (line 21) since $\tilde{Y}=U^{\left(c\right)}V^{⊤}=Q^{\left(c\right)}R^{\left(c\right)}V^{⊤}=Q^{\left(c\right)}{\left(VR^{(c)⊤}\right)}^{⊤}$.

**Algorithm 2** *compute_gradient*(*α*,*x*_*α*_,G)

**Require**: Tensor entry *x*_*α*_, $\alpha =(i_{1}\cdots i_{N})\in \Omega_{X}$, core tensor G

**Ensure**: Gradients $\frac{\partial f}{\partial u_{i_{1}}^{\left(1\right)}}$,$\frac{\partial f}{\partial u_{i_{2}}^{\left(2\right)}}$,⋯,$\frac{\partial f}{\partial u_{i_{N}}^{\left(N\right)}}$,$\frac{\partial f}{\partial G}$

1: ${\tilde{x}}_{\alpha }\leftarrow G\times {\left\{u\right\}}_{\alpha }$

2: **for** *n* = 1, ⋯, *N* **do**

3:  $\frac{\partial f}{\partial u_{i}^{\left(n\right)}}\leftarrow -(x_{\alpha }-{\tilde{x}}_{\alpha })[{\left(G\times_{-n}{\left\{u\right\}}_{\alpha }\right)}_{\left(n\right)}{]}^{⊤}+\frac{\lambda_{reg}}{|\Omega_{X}^{n,i_{n}}|}u_{i_{n}}^{\left(n\right)}$

4: **end for**

5: $\frac{\partial f}{\partial G}\leftarrow -(x_{\alpha }-{\tilde{x}}_{\alpha })\times {\left\{u\right\}}_{\alpha }^{⊤}+\frac{\lambda_{reg}}{|\Omega_{X}|}G$

6: **return** $\frac{\partial f}{\partial u_{i_{1}}^{\left(1\right)}}$,$\frac{\partial f}{\partial u_{i_{2}}^{\left(2\right)}}$,⋯,$\frac{\partial f}{\partial u_{i_{N}}^{\left(N\right)}}$,$\frac{\partial f}{\partial G}$

**Algorithm 3** *compute_gradient_opt*(*α*,*x*_*α*_,G)

**Require**: Tensor entry *x*_*α*_, $\alpha =(i_{1}\cdots i_{N})\in \Omega_{X}$, core tensor G

**Ensure**: Gradients $\frac{\partial f}{\partial u_{i_{1}}^{\left(1\right)}}$,$\frac{\partial f}{\partial u_{i_{2}}^{\left(2\right)}}$,⋯,$\frac{\partial f}{\partial u_{i_{N}}^{\left(N\right)}}$,$\frac{\partial f}{\partial G}$

1: ${\tilde{x}}_{\alpha }\leftarrow 0$

2: **for** $\forall \left(j_{1}j_{2}\ldots j_{N}\right)\in \Omega_{G}$ **do**

3:  $s_{j_{1}j_{2}\ldots j_{N}}\leftarrow g_{j_{1}j_{2}\ldots j_{N}}u_{i_{1}j_{1}}^{\left(1\right)}u_{i_{2}j_{2}}^{\left(2\right)}\ldots u_{i_{N}j_{N}}^{\left(N\right)}$

4:  ${\tilde{x}}_{\alpha }\leftarrow {\tilde{x}}_{\alpha }+s_{j_{1}j_{2}\ldots j_{N}}$

5: **end for**

6: **for** *n* = 1, …, *N* **do**

7:  $\frac{\partial f}{\partial u_{i_{n}}^{\left(n\right)}}\leftarrow -\left(x_{\alpha }-{\tilde{x}}_{\alpha }\right)\cdot Collapse\left(S,n\right)⊘u_{i_{n}}^{\left(n\right)}+\frac{\lambda_{reg}}{|\Omega_{X}^{n,i_{n}}|}u_{i_{n}}^{\left(n\right)}$

8: **end for**

9: $\frac{\partial f}{\partial G}\leftarrow -\left(x_{\alpha }-{\tilde{x}}_{\alpha }\right)\cdot S⊘G+\lambda_{reg}G$

10: **return** $\frac{\partial f}{\partial u_{i_{1}}^{\left(1\right)}}$,$\frac{\partial f}{\partial u_{i_{2}}^{\left(2\right)}}$,…,$\frac{\partial f}{\partial u_{i_{N}}^{\left(N\right)}}$,$\frac{\partial f}{\partial G}$

### S^3^CMTF-opt

There is much room for improvement in calculations of *S*^3^CMTF-base. The computational bottleneck of *S*^3^CMTF-base is *compute_gradient*. There are implicitly redundant calculations during multiple tensor-matrix products. For example, calculation of $G\times_{-n}{\left\{u\right\}}_{\alpha }$ is repeated *N* times for every execution of *compute_gradient* (Algorithm 2) in line 5 of Algorithm 1. The calculation of $G\times_{-n}{\left\{u\right\}}_{\alpha }$ for the *n*-th mode is equivalent to a special case of a well-studied operation, matricized tensor times Khatri-Rao product (MTTKRP). MTTKRP is an operation to compute **X**_(*n*)_ ⊙_∀*k* ≠ *n*_ **A**^(*k*)^ where **X**_(*n*)_ is a matricized tensor along the *n*-th mode, and ⊙ denotes the Khatri-Rao product [34]. $G\times_{-n}{\left\{u\right\}}_{\alpha }$ is equivalent to an MTTKRP **G**_(*n*)_ ⊙_∀*k* ≠ *n*_ **u**^(*k*)^ where the matrix **A**^(*k*)^ is replaced by the vector **u**^(*k*)^.

Calculating MTTKRP along all modes is known as the CP gradient problem. In *compute_gradient*, we need to calculate $G\times_{-n}{\left\{u\right\}}_{\alpha }$ for all *N* modes (line 3 of Algorithm 2), raising the special case of the CP gradient problem. To solve the particular CP gradient problem faster, we propose a method to avoid redundant computations by reusing the intermediate calculations in previous steps. Calculation of $G\times_{-n}{\left\{u\right\}}_{\alpha }$ is equivalent to a summation of $g_{j_{1}j_{2}\ldots j_{N}}u_{i_{1}j_{1}}^{\left(1\right)}\ldots u_{i_{n-1}j_{n-1}}^{\left(n-1\right)}u_{i_{n+1}j_{n+1}}^{\left(n+1\right)}\ldots u_{i_{N}j_{N}}^{\left(N\right)}$ (Eq 3) which is a product of the core value $g_{j_{1}j_{2}\ldots j_{N}}$ and *N* − 1 related factor values. Before the calculation of the CP gradient, ${\tilde{x}}_{\alpha }=G\times {\left\{u\right\}}_{\alpha }$ is calculated in line 1 of Algorithm 2. We exploit the fact that $G\times {\left\{u\right\}}_{\alpha }$ is the summation of the product $\sum_{j_{1}=1}^{J_{1}}\ldots \sum_{j_{N}=1}^{J_{N}}g_{j_{1}\ldots j_{N}}u_{i_{1}j_{1}}^{\left(1\right)}\ldots u_{i_{N}j_{N}}^{\left(N\right)}$ (Eq 4), the product of a core value and all *N* related factor values. In *S*^3^CMTF-opt, we save time by storing the intermediate calculations for ${\tilde{x}}_{\alpha }$ and reusing them.

**Definition 4**. ***(Intermediate Data)*** *When updating the factor rows for a tensor entry* $x_{\alpha =\left(i_{1}\cdots i_{N}\right)}$, *we define* (*j*_1_*j*_2_⋯*j*_*N*_)-*th element of intermediate data* S:

$$\begin{array}{c} s_{j_{1}j_{2}\cdots j_{N}}\leftarrow g_{j_{1}j_{2}\cdots j_{N}}u_{i_{1}j_{1}}^{\left(1\right)}u_{i_{2}j_{2}}^{\left(2\right)}\cdots u_{i_{N}j_{N}}^{\left(N\right)}. \end{array}$$

There is no extra time required for calculating S because S is generated while calculating ${\tilde{x}}_{\alpha }$. Lemma 1 shows that ${\tilde{x}}_{\alpha }$ is calculated by summing all entries of S.

**Lemma 1**. *For a given tensor index α*, *the estimated tensor entry* ${\tilde{x}}_{\alpha }=\sum_{j_{1}=1}^{J_{1}}\sum_{j_{2}=1}^{J_{2}}\ldots \sum_{j_{N}=1}^{J_{N}}s_{j_{1}j_{2}\ldots j_{N}}$.

*Proof*. The proof is straightforward by Eq (4).

We use S with following *Collapse* operation to calculate gradients efficiently.

**Definition 5**. ***(Collapse)*** *The Collapse operation of the intermediate tensor* S *on the n-th mode outputs a row vector defined as*:

$$\begin{array}{c} Collapse\left(S,n\right)=[\sum_{\forall \delta \in \Omega_{S}^{n,1}}s_{\delta },\sum_{\forall \delta \in \Omega_{S}^{n,2}}s_{\delta },\cdots ,\sum_{\forall \delta \in \Omega_{S}^{n,J_{n}}}s_{\delta }]. \end{array}$$

*Collapse* operation aggregates the elements of intermediate tensor S with respect to a fixed mode. We re-express the calculation of gradients for tensor factors in Eqs (8a)–(8d) in an efficient manner.

**Lemma 2**. ***(Efficient Gradient Calculation)*** *The following statements are equivalent calculations of the gradients as in* Eqs (8a)–(8d).

$$\begin{array}{cc} {\tilde{x}}_{\alpha } & \leftarrow \sum_{j_{1}=1}^{J_{1}}\sum_{j_{2}=1}^{J_{2}}\cdots \sum_{j_{N}=1}^{J_{N}}s_{j_{1}j_{2}\cdots j_{N}}, \end{array}$$ (9a)

$$\begin{array}{c} \frac{\partial f}{\partial u_{i_{n}}^{\left(n\right)}}\leftarrow -\left(x_{\alpha }-{\tilde{x}}_{\alpha }\right)\cdot Collapse\left(S,n\right)⊘u_{i_{n}}^{\left(n\right)}+\frac{\lambda_{reg}}{|\Omega_{X}^{n,i_{n}}|}u_{i_{n}}^{\left(n\right)}, \end{array}$$ (9b)

$$\begin{array}{c} \frac{\partial f}{\partial G}\leftarrow -\left(x_{\alpha }-{\tilde{x}}_{\alpha }\right)\cdot S⊘G+\lambda_{reg}G. \end{array}$$ (9c)

*where α* = (*i*_1_ *i*_2_⋯*i*_*N*_), *and* ⊘ *denotes element-wise division*.

*Proof*. In Lemma 1, Eq (9a) is proved. To prove the equivalence of Eq (9b) and the Eq (8a), it suffices to show ${\left[{\left(G\times_{-n}{\left\{u\right\}}_{\alpha }\right)}_{\left(n\right)}\right]}^{⊤}=Collapse\left(S,n\right)⊘u_{i_{n}}^{\left(n\right)}$ We use Eq (3) for the proof. $\alpha =\left(i_{1}\ldots i_{N}\right)\in \Omega_{X}$ and $\delta =\left(j_{1}\ldots j_{N}\right)\in \Omega_{G}^{n,k}$.

$$\begin{array}{c} \begin{array}{cc} {\left[{\left(G\times_{-n}{\left\{u\right\}}_{\alpha }\right)}_{\left(n\right)}\right]}_{k}^{⊤} & =\sum_{\forall \delta \in \Omega_{G}^{n,k}}g_{\delta }u_{i_{1}j_{1}}^{\left(1\right)}\cdots u_{i_{n-1}j_{n-1}}^{\left(n-1\right)}u_{i_{n+1}j_{n+1}}^{\left(n+1\right)}\cdots u_{i_{N}j_{N}}^{\left(N\right)}\\ & =\sum_{\forall \delta \in \Omega_{G}^{n,k}}g_{\delta }u_{i_{1}j_{1}}^{\left(1\right)}\cdots u_{i_{n-1}j_{n-1}}^{\left(n-1\right)}u_{i_{n}k}^{\left(n\right)}u_{i_{n+1}j_{n+1}}^{\left(n+1\right)}\cdots u_{i_{N}j_{N}}^{\left(N\right)}/u_{i_{n}k}^{\left(n\right)}\\ & =\sum_{\forall \delta \in \Omega_{S}^{n,k}}s_{\delta }/u_{i_{n}k}^{\left(n\right)}\\ & =\frac{{\left[Collapse\left(S,n\right)\right]}_{k}}{u_{i_{n}k}^{\left(n\right)}}\\ & ={\left[Collapse\left(S,n\right)⊘u_{i_{n}}^{\left(n\right)}\right]}_{k}. \end{array} \end{array}$$

Next, to show the equivalence of Eq (9c) and the second equation of Eq (8), it suffices to show $1\times {\left\{u\right\}}_{\alpha }^{⊤}=S⊘G$.

$$\begin{array}{c} \begin{array}{cc} {\left[1\times {\left\{u\right\}}_{\alpha }^{⊤}\right]}_{\gamma =(l_{1}l_{2}\cdots l_{N})} & =u_{i_{1}l_{1}}^{\left(1\right)}u_{i_{2}l_{2}}^{\left(2\right)}\cdots u_{i_{N}l_{N}}^{\left(N\right)}\\ & =g_{\gamma }u_{i_{1}l_{1}}^{\left(1\right)}\cdots u_{i_{N}l_{N}}^{\left(N\right)}/g_{\gamma }\\ & =s_{\gamma }/g_{\gamma }\\ & ={\left[S⊘G\right]}_{\gamma }. \end{array} \end{array}$$

*S*^3^CMTF-opt replaces *compute_gradient* (Algorithm 2) of *S*^3^CMTF-base with *compute_gradient_opt* (Algorithm 3), the time-optimized alternative. We prove that the new calculation scheme is faster than the previous one.

**Lemma 3**. *compute_gradient_opt is faster than compute_gradient*. *The theoretical time complexity of compute_gradient is* $O(N^{2}J^{N})$ *and the time complexity of compute_gradient_opt is* $O(NJ^{N})$ *where J*_1_ = *J*_2_ = ⋯ = *J*_*N*_ = *J*.

*Proof*. We assume that *I*_1_ = *I*_2_ = ⋯ = *I*_*N*_ = *I* for brevity. First, we calculate the time complexity of *compute_gradient* (Algorithm 2). Given a tensor index *α*, computing ${\tilde{x}}_{\alpha }$ (line 1 of Algorithm 2) takes $O(NJ^{N})$. Computing ($G\times_{-n}{\left\{u\right\}}_{\alpha }$) (line 3) takes $O(NJ^{N})$. Thus, aggregate time for calculating the row gradient for all modes (lines 2-4) takes $O(N^{2}J^{N})$. Calculating $\left(x_{\alpha }-{\tilde{x}}_{\alpha }\right)\times {\left\{u\right\}}_{\alpha }^{⊤}$ (line 5) takes $O(NJ^{N})$. In total, *compute_gradient* takes $O(N^{2}J^{N})$ time. Next, we calculate the time complexity of *compute_gradient_opt* (Algorithm 3). Computing an entry of intermediate data S (line 3 of Algorithm 3) takes O(N). Aggregate time for getting S (lines 2-5) is $O(NJ^{N})$ since $|\Omega_{G}|=O\left(J^{N}\right)$. Calculating row gradient for all modes (lines 6-8) takes $O(NJ^{N})$ since *Collapse* operation takes $O(J^{N})$. Calculating gradient for core tensor (line 9) takes $O(J^{N})$. In total, *compute_gradient_opt* takes $O(NJ^{N})$ time.

### Analysis

We analyze the proposed method in terms of time complexity per iteration. For simplicity, we assume that *I*_1_ = *I*_2_ = ⋯ = *I*_*N*_ = *I*, and *J*_1_ = *J*_2_ = ⋯ = *J*_*N*_ = *J*. Table 3 summarizes the time complexity (per iteration) and memory usage of *S*^3^CMTF and other methods. Note that the memory usage refers to the auxiliary space for temporary variables used by a method.

**Table 3:** Comparison of time complexity (per iteration) and memory usage of our proposed *S*^3^CMTF and other CMTF algorithms. *S*^3^CMTF-opt shows the lowest time complexity and *S*^3^CMTF-base shows the lowest memory usage. For simplicity, we assume that all modes are of size *I*, of rank *J*, and an *I* × *K* matrix is coupled to one mode. *P* is the number of parallel cores. (* indicates the lowest time or memory).

	Time complexity (per iter.)	Memory usage
**S^3^CMTF-base**	$O(|\Omega_{X}|N^{2}J^{N}/P+|\Omega_{Y}|J/P)$	O(PJ)*
**S^3^CMTF-opt**	$O(|\Omega_{X}|NJ^{N}/P+|\Omega_{Y}|J/P)$*	$O(PJ^{N})$
CMTF-Tucker-ALS	$O(NI^{N-1}J^{2}+NI^{2}J^{N-1}+I^{2}K)$	$O(IJ^{N-1})$
CMTF-OPT	$O(|\Omega_{X}|NJ+NI^{N-1}J+IJK)$	$O(I^{N-1}J+JK)$

**Lemma 4**. *The time complexity (per iteration) of S*^3^CMTF-*base is* $O(|\Omega |N^{2}J^{N}/P+|\Omega_{Y}|J/P)$ *and the time complexity (per iteration) of S*^3^CMTF-*opt is* $O(|\Omega |NJ^{N}/P+|\Omega_{Y}|J/P)$ *where P denotes the number of parallel cores*.

*Proof*. First, we check the time complexity of *S*^3^CMTF-base. When a tensor index *α* is picked in the inner loop (line 4 of Algorithm 1), calculating gradients with respect to tensor factors (line 5) takes $O(N^{2}J^{N})$ as shown in Lemma 3. Updating factor rows (line 6) takes O(NJ), and updating core tensor (line 7) takes $O(J^{N})$. If a coupled matrix index *β* is picked (line 9), calculating ${\tilde{y}}_{\beta }$ (line 10) takes O(J). Calculating and updating the factor rows corresponding to coupled matrix entry (lines 10-12) take O(J). All calculations except updating core tensor (line 7) are conducted in parallel. Finally, for all $\alpha \in \Omega_{X}$ and *β* ∈ Ω_**Y**_, *S*^3^CMTF-base takes $O(|\Omega_{X}|N^{2}J^{N}/P+|\Omega_{Y}|J/P)$ for one iteration. *S*^3^CMTF-opt uses *compute_gradient_opt* instead of *compute_gradient* in line 5 of Algorithm 1, whose time complexity is shown in Lemma 3. Overall running time per iteration for *S*^3^CMTF-opt is $O(|\Omega_{X}|NJ^{N}/P+|\Omega_{Y}|J/P)$.

## Experiments

In this and the next sections, we experimentally evaluate *S*^3^CMTF. Especially, we answer the following questions.

**Q1**: **Performance** How accurate and fast is *S*^3^CMTF compared to competitors?

**Q2**: **Scalability** How do *S*^3^CMTF and other methods scale in terms of dimensionality, the number of observed entries, and the number of cores?

**Q3**: **Discovery** What are the discoveries of applying *S*^3^CMTF on real-world data?

The source codes of our method and datasets used in this paper are available at https://datalab.snu.ac.kr/S3CMTF.

### Experimental settings

#### Data

Table 4 shows the data we used in our experiments. We use three real-world datasets, MovieLens (http://grouplens.org/datasets/movielens/10m), Netflix (http://www.netflixprize.com), and Yelp (http://www.yelp.com/dataset_challenge), as well as synthetic data to evaluate *S*^3^CMTF. Each entry of the real-world datasets represents a rating, which consists of (user, ‘item’, time; rating) where ‘item’ indicates ‘movie’ for MovieLens and Netflix, and ‘business’ for Yelp. We use (movie, genre) and (movie, year) as coupled matrices for MovieLens and Netflix, respectively. We use (user, user) friendship matrix, (business, category) and (business, city) matrices for Yelp. Particularly for scalability experiments, we generate 3-mode synthetic random tensors with dimensionality *I* and corresponding coupled matrices to observe speed property while size is varying. We vary *I* in the range of 1K∼100M and the number of tensor entries in the range of 1K∼100M. We set the number of entries as $|\Omega_{Y}|=\frac{1}{10}\left|\Omega_{X}\right|$ for synthetic coupled matrices. We generated observed indices randomly, and their entries to follow uniform random distribution between 0 and 1.

**Table 4:** Summary of the data used for experiments. ‘K’ means thousand, and ‘M’ million. Tensors and matrices of density 1 are fully observed.

Name	Data	Dimensionality	# entries	Density
MovieLens	User-Movie-Time	71K-11K-157	10M	∼10^−4^
	Movie-Genre	20	214K	1
Netflix	User-Movie-Time	480K-18K-74	100M	∼10^−4^
	Movie-Yearmonth	110	2M	1
Yelp	User-Business-Time	1M-144K-149	4M	∼10^−7^
	User-User	1M	7M	∼10^−4^
	Business-Category	1K	172M	1
	Business-City	1K	126M	1
Synthetic	3-mode tensor	1K∼100M	1K∼100M	10^−20 *to* −3^
	Matrix	1K∼100M	1K∼100M	10^−11 *to* −4^

#### Measure

We use test RMSE as the measure for tensor reconstruction error.

$$\begin{array}{c} \text{test}\;\text{RMSE}=\sqrt{\frac{1}{|\Omega_{test}|}\sum_{\forall \alpha \in \Omega_{test}}{\left(x_{\alpha }-{\tilde{x}}_{\alpha }\right)}^{2}} \end{array}$$

where Ω_*test*_ is the index set of the test data tensor, *x*_*α*_ stands for each test tensor entry, and ${\tilde{x}}_{\alpha }$ is the corresponding reconstructed value.

#### Methods

For fair comparison, we compare single core run of *S*^3^CMTF-base and *S*^3^CMTF-opt with other single machine CMTF methods: CMTF-Tucker-ALS and CMTF-OPT (described in Section). To examine multi-core performance, we run two versions of *S*^3^CMTF-opt: *S*^3^CMTF-opt1 (1 core), and *S*^3^CMTF-opt20 (20 cores). We exclude distributed CMTF methods [25, 26, 28] since they are designed for Hadoop with multiple machines, and thus take too much time for single machine environment. For example, [17] reported that HaTen2 [26] takes 10,700s to decompose 4-way tensor with *I* = 10*K* and $|\Omega_{X}|=100K$, which is almost 7,000× slower than our single machine implementation of *S*^3^CMTF-opt. For CMTF-Tucker-ALS, we implemented a C++ version based on Tucker-MET [20], and for CMTF-OPT, we implemented a C++ version of CMTF-OPT [12]. Our implementation for CMTF-OPT solves Eq (6) by sparse matrix operations. We implement *S*^3^CMTF with C++. For all of our C++ implementations, we used C++11 with O2 flag. We used Armadillo 7.700 with LAPACK 3.7.0 and BLAS 3.7.0 for matrix operations such as eigenvector calculations. We used OpenMP 4.0 library for multi-core parallelization of *S*^3^CMTF.

We conduct all experiments on a machine equipped with Intel Xeon E5-2630 v4 2.2GHz CPU and 256GB RAM. We mark out-of-memory (O.O.M.) error when the memory usage exceeds the limit.

#### Hyperparameters

We set pre-defined hyperparameters that resulted in the best reconstruction error on a 10% validation set by random grid search: tensor rank *J*, regularization factor λ_*reg*_, λ_*m*_, the initial learning rate *η*_0_, and decay rate *μ*. We set λ_*reg*_ to 0.1, λ_*m*_ = 10, and *μ* = 0.1 for all datasets. For rank and initial learning rate, MovieLens: *J* = 12, *η* = 0.001, Netflix: *J* = 11, *η* = 0.001, and Yelp: *J* = 10, *η* = 0.0005. For synthetic datasets, we use *J* = 10 for all experiments.

### Performance of S^3^CMTF

**Fig 4:**
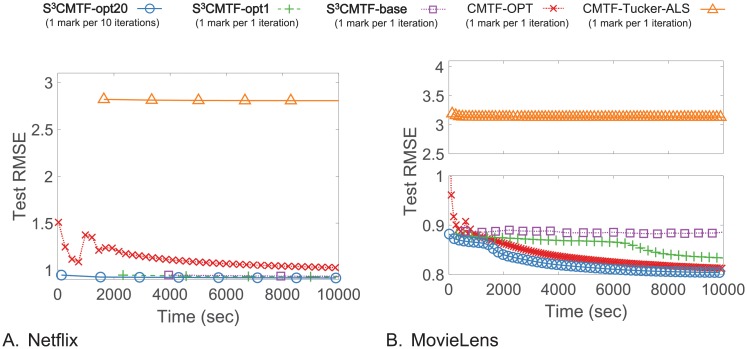
Test RMSE of *S*^3^CMTF and other CMTF methods over iterations. *S*^3^CMTF-opt20 shows the best convergence rate and accuracy.

We observe the performance of *S*^3^CMTF to answer Q1. As seen in Figs 1B and 4, *S*^3^CMTF converges faster to the optimum with the lowest test error than existing methods with the following details.

#### Accuracy

We divide each data tensor into 80%/20% for train/test sets. Specifically, 80% of the tensor entries are regarded as the train set and remaining 20% as the test set. The lower error for a same elapsed time implies the better accuracy and faster convergence. Figs 1B and 4 show the changes of test RMSE of each method on three datasets over elapsed time which are the answers for Q1. *S*^3^CMTF achieves the lowest error compared to others for the same elapsed time. For Yelp, CMTF-Tucker-ALS yielded an O.O.M. error. *S*^3^CMTF-opt20 achieves the lowest error 1.253, 0.9147, and 0.8037 while the best competing method, CMFT-OPT, gives the error 1.370, 1.018, and 0.8125 for Yelp, Netflix, and MovieLens datasets, respectively. Note that the competing method CMFT-Tucker-ALS gives either an out of memory error or results in the highest error rate.

#### Running time

**Fig 5:**
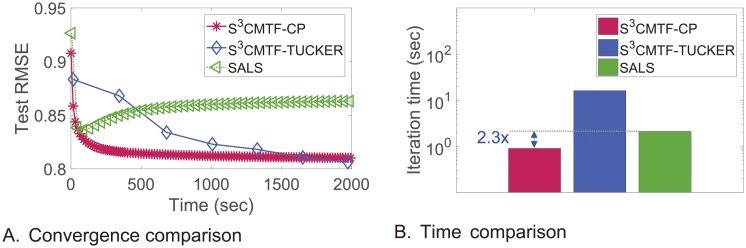
Comparison with SALS-single for movieLens dataset. We compare two non-coupled version of *S*^3^CMTF, *S*^3^CMTF-CP-opt and *S*^3^CMTF-TUCKER-opt with the parallel CP decomposition method, SALS-single. For (a), we set 1 mark per 20 iterations for clarity. (a) *S*^3^CMTF converges faster to a lower error than SALS does. (b) *S*^3^CMTF-CP-opt is 2.3× faster than SALS-single.

We compare our method with the multi-core version of SALS-single [30], a parallel CP decomposition algorithm, to demonstrate the high performance of *S*^3^CMTF compared to the state-of-the-art decomposition algorithms. We used non-coupled CP version of our method, *S*^3^CMTF-CP-opt, by setting G to be hyper-diagonal and not coupling any matrices. Fig 5 shows that *S*^3^CMTF is better than SALS-single in terms of both error and time for MovieLens dataset. *S*^3^CMTF-TUCKER explicitly denotes *S*^3^CMTF-opt for Tucker model.

### Scalability analysis

We present scalability of our proposed *S*^3^CMTF and competitors to answer Q2, in terms of two aspects: data scalability and parallel scalability. We use synthetic data of varying size for evaluation. As a result, we show the running time (for one iteration) of *S*^3^CMTF follows our theoretical analysis in Section.

#### Data scalability

**Fig 6:**
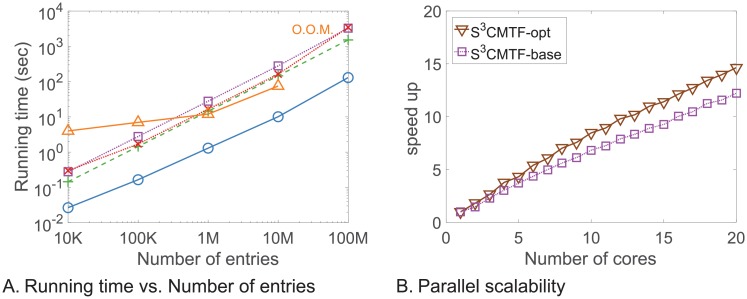
Comparison of scalability. (a) *S*^3^CMTF shows linear scalability as the number of entries increases. (b) *S*^3^CMTF-base and *S*^3^CMTF-opt show linear *speed up* as the number of cores grows. O.O.M.: out of memory error.

The time complexity of CMTF-Tucker-ALS and CMTF-OPT have $O(NI^{N-1}J^{2})$ and $O(NI^{N-1}J)$ as their dominant terms, respectively. In contrast, *S*^3^CMTF exploits the sparsity of input data, and has the time complexity linear to the number of entries ($|\Omega_{X}|$, |Ω_**Y**_|) and is independent of the dimensionality (*I*) as shown in Lemma 4. Figs 1A and 6A show that the running time (for one iteration) of *S*^3^CMTF on real world data sets follows our theoretical analysis in Section. First, we fix $|\Omega_{X}|$ to 1M and |Ω_**Y**_| to 100K, and vary dimensionality *I* from 1K to 100M. Fig 1A shows the running time (for one iteration) of all methods with *J* = 10. Note that all of our proposed methods achieve constant running time as dimensionality increases because they exploit the sparsity of data by updating factors related to only observed data entries. However, CMTF-Tucker-ALS and CMTF-OPT show exponentially increasing running time, and CMTF-OPT shows O.O.M. when *I* = 10*M*. Next, we investigate the data scalability over the number of entries as shown in Fig 6A. We fix *I* to 10K and raise $|\Omega_{X}|$ from 10K to 100M. CMTF-Tucker-ALS shows O.O.M. when $|\Omega_{X}|=100M$, and CMTF-OPT shows near-linear scalability. Focusing on the results of *S*^3^CMTF, all three versions of our approach show linear relation between running time and $|\Omega_{X}|$.

#### Parallel scalability

We conduct experiments to examine parallel scalability of *S*^3^CMTF on shared memory systems. For measurement, we define *speed up* as *(iteration time on 1 core)*/*(iteration time)*. Fig 6B shows the linear *speed up* of *S*^3^CMTF-base and *S*^3^CMTF-opt. The slope of the parallel scalability curve is not one (perfectly parallelizable) since the growing number of cores leads to the concurrent read accesses to memory, which leads to conflicts. *S*^3^CMTF-opt shows higher *speed up* than *S*^3^CMTF-base because it reduces reading accesses for core tensor by utilizing intermediate data.

## Discovery

In this section, we use *S*^3^CMTF for mining real-world data, Yelp, to answer the question Q3 in the beginning of the previous section. First, we demonstrate that *S*^3^CMTF has better discernment for business entities compared to the naive decomposition method by jointly capturing spatial and categorical prior knowledge. Second, we show how *S*^3^CMTF is possibly applied to the real recommender systems. It is an open challenge to jointly capture the spatio-temporal context along with user preference data [35]. We exemplify a personal recommendation for a specific user. For discovery, we use the total Yelp data tensor along with coupled matrices as explained in Table 4. For better interpretability, we found a non-negative factorization by applying projected gradient method [36]. An orthogonality condition is not imposed to keep non-negativity, and each column of factors is normalized.

### Discovery

**Fig 7:**
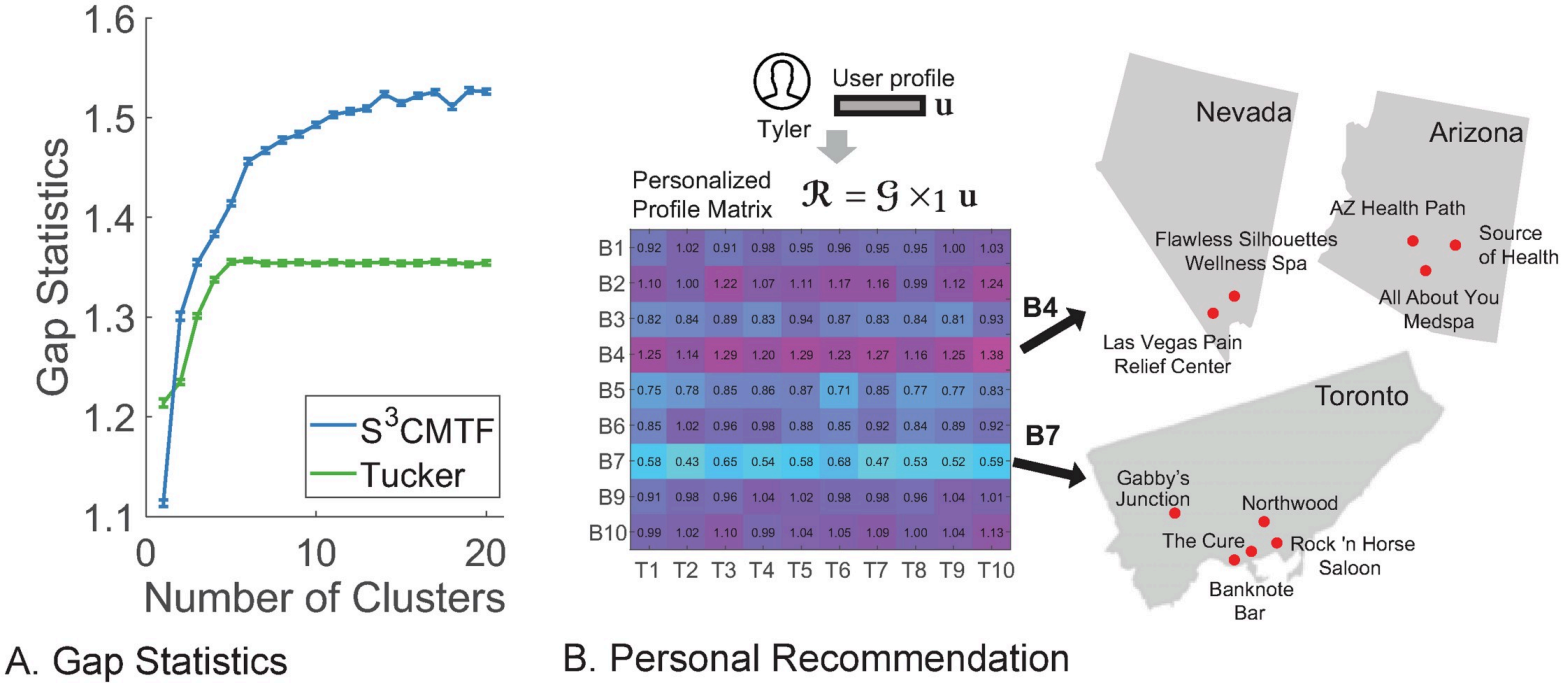
(a) Gap statistics on **U**^(2)^ of *S*^3^CMTF and the Tucker decomposition for Yelp dataset. *S*^3^CMTF outperforms the naive Tucker decomposition for its clustering ability. (b) Visualization of the personal recommendation scenario.

First, we compare discernment by *S*^3^CMTF and the Tucker decomposition. We use the business factor **U**^(2)^. Fig 7A shows the gap statistic values of clustering business entities with k-means clustering algorithm. Gap statistic is a theoretical tool to measure separability between k-means clusters [37]. A higher gap statistic means higher separability between clusters. *S*^3^CMTF shows higher gap statistic values compared to the Tucker decomposition which means *S*^3^CMTF outperforms the naive Tucker decomposition for entity clustering with respect to the gap statistic.

As the difference between *S*^3^CMTF and the Tucker decomposition is in the existence of coupled matrices, the high performance of *S*^3^CMTF is attributed to the unified factorization using spatial and categorical data as prior knowledge. Table 5 shows the found clusters of business entities. Note that each cluster represents a certain combination of spatial and categorical characteristics of business entities.

**Table 5:** Clustering results on business factor U^(2)^ found by *S*^3^CMTF. We found dominant spatial and categorical characteristics from each cluster. Businesses in a same cluster tend to be in adjacent cities and are included in similar categories.

Cluster	Location / Category	Top-10 Businesses
**C1**	Las Vegas, US/ Travel & Entertainment	Nocturnal Tours, Eureka Casino, Happi Inn, Planet Hollywood Poker Room, Circus Midway Arcade, etc.
**C2**	Arizona, US/ Real estate & Home services	ENMAR Hardwood Flooring, Sprinkler Dude LLC, Eklund Refrigeration, NR Quality Handyman, The Daniel Montez Real Estate Group, etc.
**C11**	Ontario, Canada/ Restaurants & Deserts	Jyuban Ramen House, Tim Hortons, Captain John Donlands Fish and Chips, Cora’s Breakfast & Lunch, Pho Pad Thai, etc.
**C17**	Ohio, US/ Food & Drinks	ALDI, Pulp Juice and Smoothie Bar, One Barrel Brewing, Wok N Roll Food Truck, Gas Pump Coffee Company, etc.

### User-specific recommendation

Commercial recommendations are one of the most important applications of factorization models [4, 9]. Here we illustrate how factor matrices are used for personalized recommendations with a real example. Fig 7B shows the process for recommendation. Below, we illustrate the process in detail.

- An example user Tyler has a factor vector **u**, namely user profile, which has been calculated by previous review histories.
- We then calculate the personalized profile matrix $R=G\times_{1}u\left(\in R^{J_{2}\times J_{3}}\right)$. R measures the amount of interaction of user profile with business and time factors.
- Norm values of rows in R indicate the influence of latent business concepts on Tyler. Dominant and weak concepts are found based on the calculated norm values. In the example, B4 is the dominant, and B7 is the weak latent concept.
- We inspect the corresponding columns of business factor matrix **U**^(2)^ and find relevant business entities with high values for the found concepts (B4 and B7).

We found both strong and weak entities by the above process. The strong and weak entities provide recommendation information by themselves in the sense that the probability of the user to like strong and weak entities are high and low, respectively, and they also give extended user preference information. For example, strong entities for Tyler are related to ‘spa & health’ and located in neighborhood cities of Arizona, US. Weak entities are related to ‘grill & restaurants’ and located in Toronto, Canada. The captured user preference information potentially makes commercial recommender systems interpretable with additional user-specific information such as address, current location among others.

## Conclusion

We propose *S*^3^CMTF, a fast, accurate, and scalable CMTF method. *S*^3^CMTF provides up to 930× faster running times and the best accuracy by sparse CMTF with carefully derived update rules, lock-free parallel SGD, and reusing intermediate computation results. *S*^3^CMTF shows linear scalability for the number of data entries and parallel cores. Moreover, we show the usefulness of *S*^3^CMTF for cluster analysis and recommendation by applying *S*^3^CMTF to real-world Yelp data. For future improvements, applying recent achievements in the literature to improve CP gradient algorithm [38, 39] to our method is possible. Also, future works include extending the method to a distributed setting.
